# Supplementary material for: αN-Acetyl β-Endorphin Is an Endogenous Ligand of σ1Rs That Regulates Mu-Opioid Receptor Signaling by Exchanging G Proteins for σ2Rs in σ1R Oligomers
Source: Int J Mol Sci. 2022 Dec 29;24(1):582. doi: 10.3390/ijms24010582 (PMC9820303; doi:10.3390/ijms24010582)
Supplement: Supplementary file 1 [file ijms-24-00582-s001.zip › ijms-2004912-supplementary.pdf]

$^{\alpha}$*N*-acetyl β-endorphin is an endogenous ligand of σ1Rs that regulates mu-opioid receptor signaling by exchanging G proteins for σ2Rs in σ1R oligomers

Javier Garzón-Niño, Elsa Cortés-Montero, María Rodríguez-Muñoz, Pilar Sánchez-Blázquez

Neuropharmacology, Cajal Institute, Department of Translational Neuroscience, CSIC, Madrid, Spain.

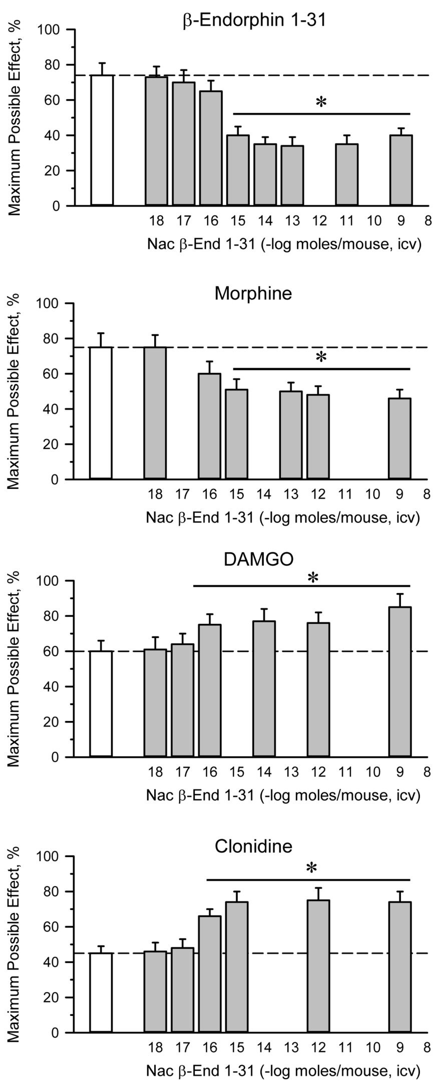

Figure S1. The effect of $^{\alpha}N$-acetyl β-End 1-31 on opioid- and nonopioid-induced analgesia. The mice were icv-injected with increasing doses of $^{\alpha}N$-acetyl β-End 1-31 20 min before icv treatment with β-End 1-31 (0.5 nmol), morphine (10 nmol), DAMGO (0.1 nmol) and clonidine (150 nmol). Analgesia was determined in the thermal tail-flick test at the corresponding analgesic peak effect intervals, 15 minutes after DAMGO, 30 minutes after β-End 1-31 and morphine, and 45 minutes after clonidine administration. The bars are the mean ± SD of the data from six mice. * Indicates a significant difference from the control group (white bar) which, received saline instead of the analgesic compound; ANOVA followed by the Holm-Sidak multiple comparisons test, $p < 0.05$.

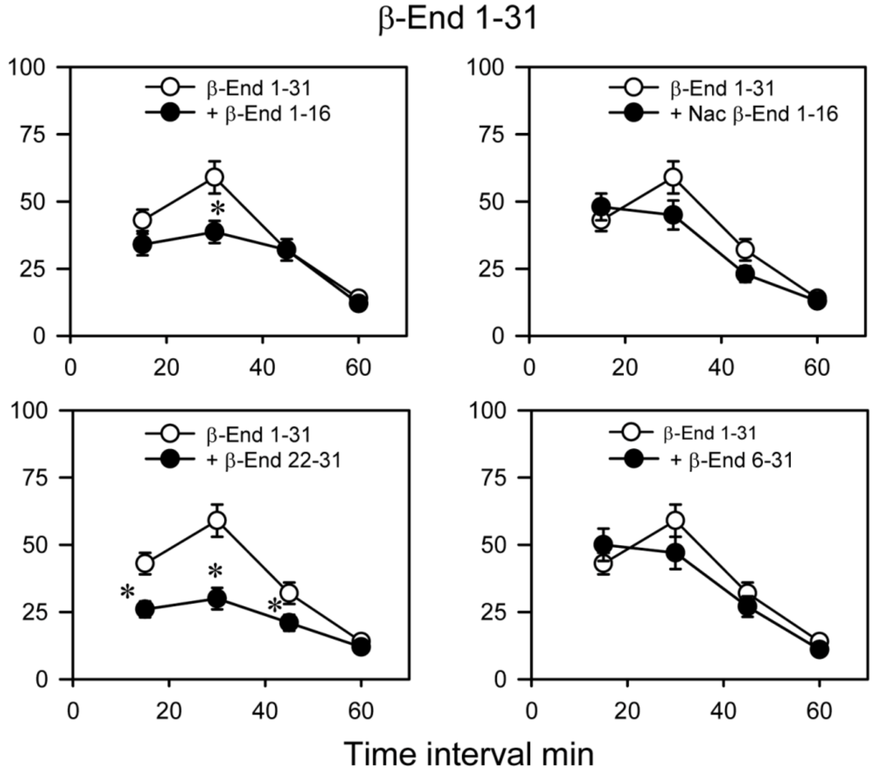

Figure S2. The effect of nonphysiological β-End 1-31 fragments on the analgesia produced by icv β-End 1-31. The mice were icv-injected with 1 nmol of selected sequences of β-End 1-31 20 min before icv treatment with 0.5 nmol of the complete β-End 1-31 peptide sequence. Analgesia was determined at the indicated post-administration intervals. The points are the mean ± SD of the pooled data from six mice. For every postopioid interval, * indicates that the β-End 1-31 sequence being evaluated significantly altered the analgesic response of the mice to β-End 1-31. ANOVA followed by the Holm-Sidak multiple comparisons test, $p < 0.05$.

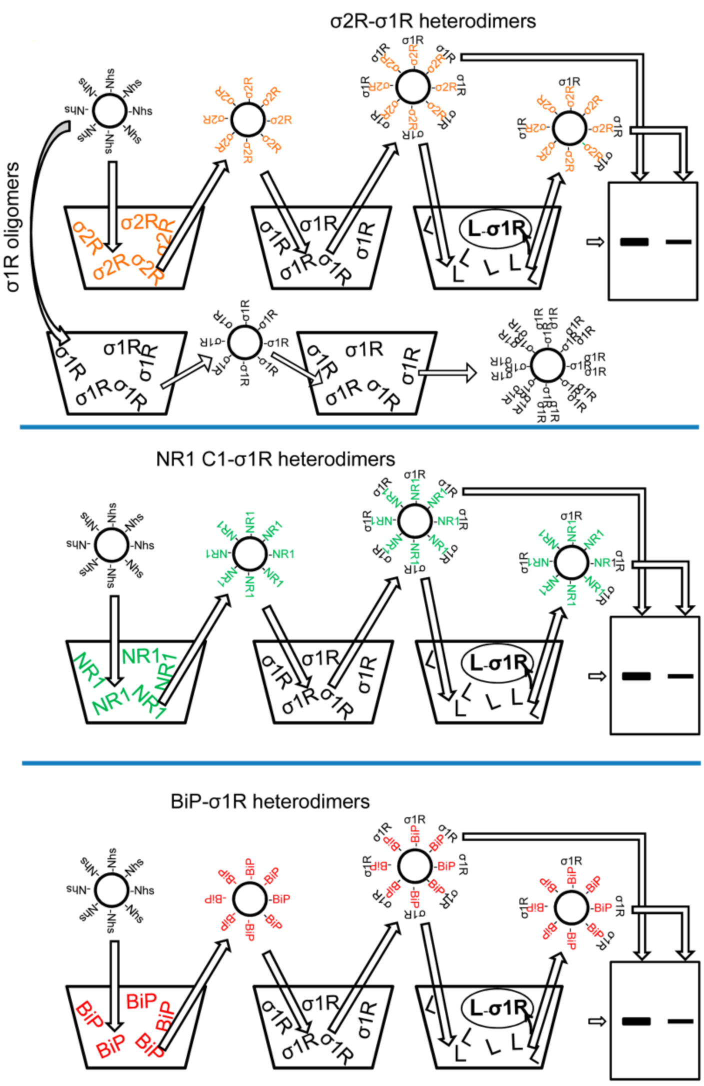

Figure S3. ***In vitro*** **assay protocols.** Recombinant σ2Rs, the cytosolic region of NMDAR NR1 C1 subunits, or BiP were covalently attached to agarose. The agarose complexes were incubated with excess σ1Rs in the absence (control) and presence of increasing concentrations of the substances under study. The unbound σ1Rs were washed out, and the σ1Rs that remained attached to the agarose-bound proteins were then evaluated by SDS-PAGE and immunoblotting. An identical procedure was used to obtain *in vitro* σ1R oligomers that were bound to agarose-σ1Rs Further details are provided in Methods.

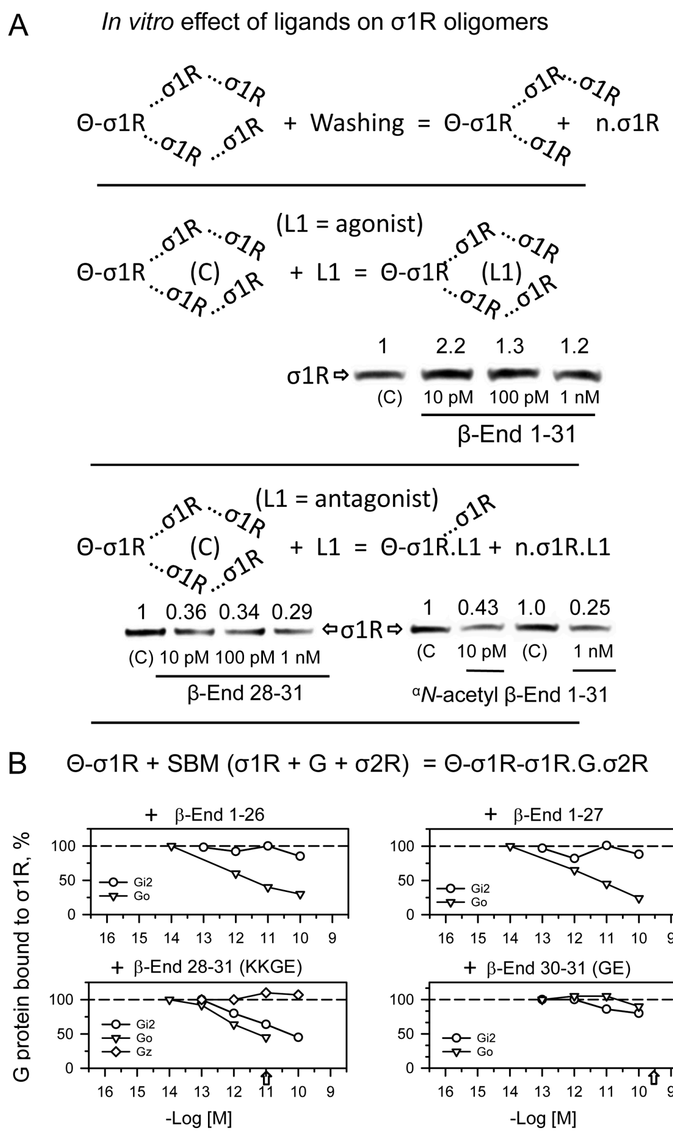

Figure S4. ***In vitro*** **made σ1R oligomers. A)** Effect of σ1R ligands on the formation of σ1R oligomers. Agarose (Θ)-attached σ1Rs were incubated for 30 min in the presence of recombinant free σ1Rs (100 nM) in 300 μL of 50 mM Tris-HCl (pH 7.4), 0.2% CHAPS, and 2.5 mM $CaCl_2$. After removing unbound σ1R proteins, the assays were performed in the absence (control) or presence of increasing concentrations of σ1R ligands. The agarose-bound σ1R protein complexes were obtained by centrifugation, washed three times, solubilized in 2x Laemmli buffer containing β-mercaptoethanol, resolved by SDS-PAGE, and analyzed by Western blotting (for more details see Methods). Representative blots are shown. **B)** Effect of ligands on the association of endogenous G proteins in solubilized mouse cortical brain membranes (SBM) with Θ-σ1R oligomers made *in vitro*. The Θ-σ1Rs were incubated with SBM in the absence and presence of increasing concentrations of physiological β-End 1-31 derivatives. The G proteins that remained bound to Θ-σ1R oligomers were determined as in Figure 4C. The arrows indicate an estimated affinity of the ligand for σ1Rs, as determined through *in vitro* assays.

Effect of σ1R antagonist S1RA and ᵅN-acetyl β-End 1-31 on σ1R E102Q associations with NR1 C1 and σ2R.

Effect of σ1R agonist PRE084 and β-End 1-31 on σ1R E102Q associations with NR1 C1 and σ2R.

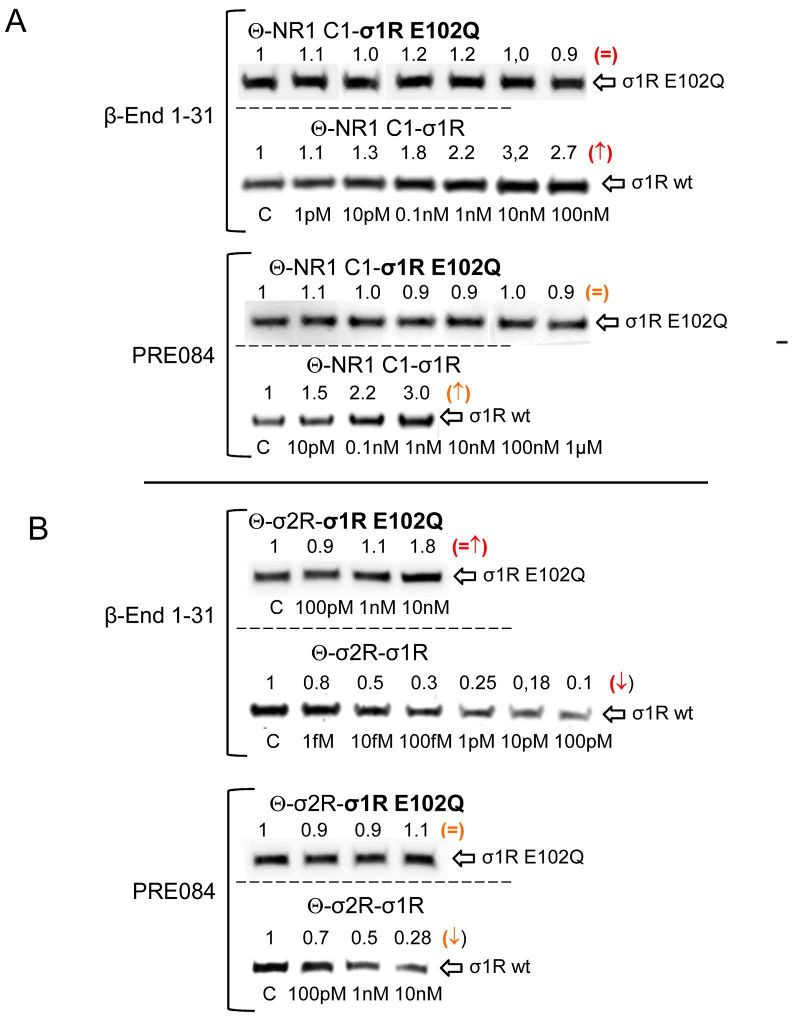

Figure S5. Effect of σ1R antagonist S1RA and ᵅ*N*-acetyl β-End 1-31 on the association of σ1R E102Q mutant with NR1 C1 and σ2R. **(A)** Agarose (Θ)-attached NR1 C1 proteins were incubated for 30 min in the presence of 100 nM recombinant σ1R human mutant E102Q or σ1R wild-type (WT) proteins in 300 μL of 50 mM Tris-HCl (pH 7.4), 0.2% CHAPS, and 2.5 mM $CaCl_2$. After the unbound σ1R proteins were removed, the effects of increasing concentrations of S1RA and ᵅ*N*-acetyl β-End 1-31 on NR1 C1-σ1R E102Q/WT associations were studied. **(B)** HaloLink resin (Θ) (G1912, Promega)-attached σ2R proteins were coupled to 100 nM recombinant σ1R human mutant E102Q or σ1R wild-type (WT) proteins to study the effect of increasing concentrations of S1RA and ᵅ*N*-acetyl β-End 1-31 on NR1 C1-σ1R E102Q/WT associations. The assays were repeated twice and the results were comparable. Recombinant WT σ1R and E102Q mutant bind to NHS-activated agarose–C0-C1-C2 region of the NR1 subunit, but not to inactivated NHS-Sepharose® (negative control) (Rodríguez-Muñoz et al., *Int J. Mol. Sci*. **2020**, *21,* 7229).

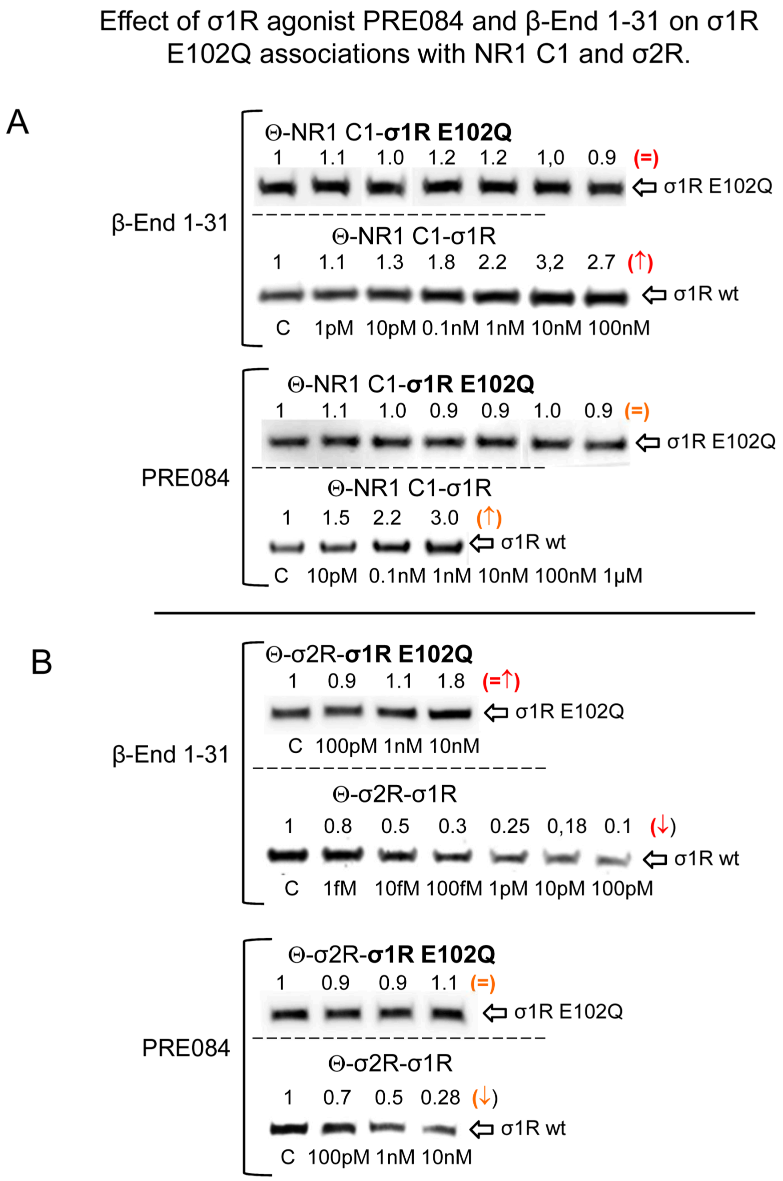

Figure S6. Effect of σ1R agonist PRE084 and β-End 1-31 on the association of σ1R E102Q mutant with NR1 C1 and σ2R. **(A)** Agarose (Θ)-attached NR1 C1 proteins and, **(B)** HaloLink resin (Θ)-attached σ2R proteins, were coupled to 100 nM recombinant σ1R human mutant E102Q or σ1R wild-type (WT) proteins. The effects of increasing concentrations of PRE084 and β-End 1-31 on these associations were studied. Details as in Figure S5.

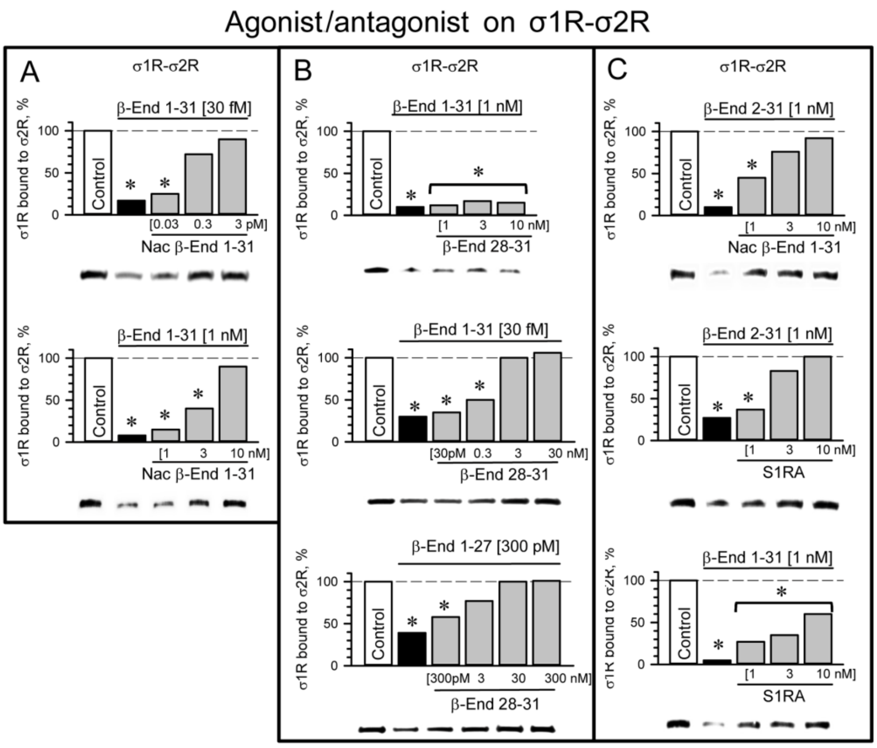

Figure S7. Effect of σ1R agonist/antagonist ligands on σ1R-σ2R heterodimers. (**A-C**) Agarose (Θ)-attached σ2Rs captured recombinant σ1Rs. After removing unbound σ1R proteins, the Θ-σ2R.σ1R complexes were incubated with a fixed concentration of a selected L1 ligand (indicated in brackets) in the absence and presence of increasing concentrations of competing σ1R ligands as indicated. The σ1Rs that remained attached to the Θ-σ2Rs were evaluated by Western blotting. * Significant differences with respect to the control group that was incubated without L1 ligands. ANOVA followed by the Holm-Sidak multiple comparisons test, $p < 0.05$. For details, see Methods and Figure 6A. Representative blots are shown.

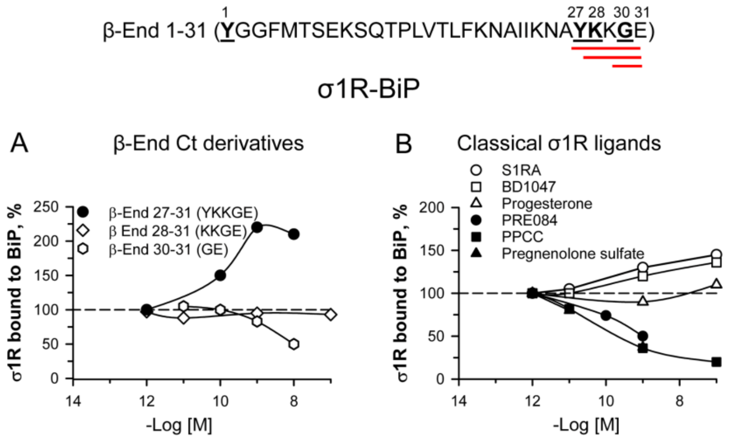

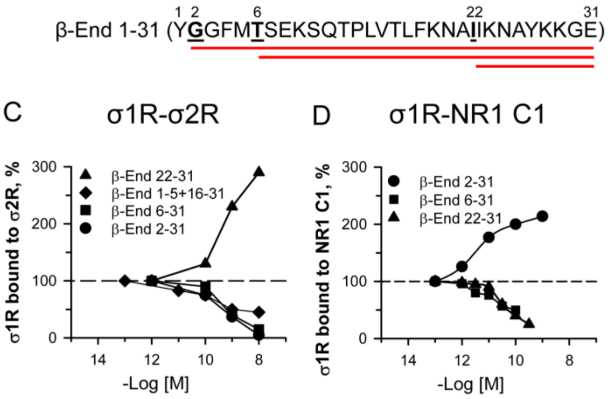

Figure S8. Effect of the physiological and nonphysiological sequences of β-End 1-31 and classical σ1R ligands on BiP-σ1R, σ2R-σ1R and NR1 C1-σ1R associations. Agarose-BiP was loaded with σ1Rs, and after the unbound σ1Rs were removed, the agarose-BiP-σ1R complexes were incubated in the absence (control) and presence of increasing concentrations of **(A)** β-End 1-31 C-terminal physiological derivatives and **(B)** classical σ1R ligands. **C)** Agarose-σ2R loaded with σ1Rs was incubated in the absence (control) and with nonphysiological derivatives of β-End 1-31. **D)** Agarose-NR1 C1 loaded with σ1Rs were incubated in the absence (control) and with nonphysiological derivatives of β-End 1-31. **A-D)** The σ1Rs that remained attached to the agarose complexes were evaluated by Western blotting. The details are shown in Figure 6.

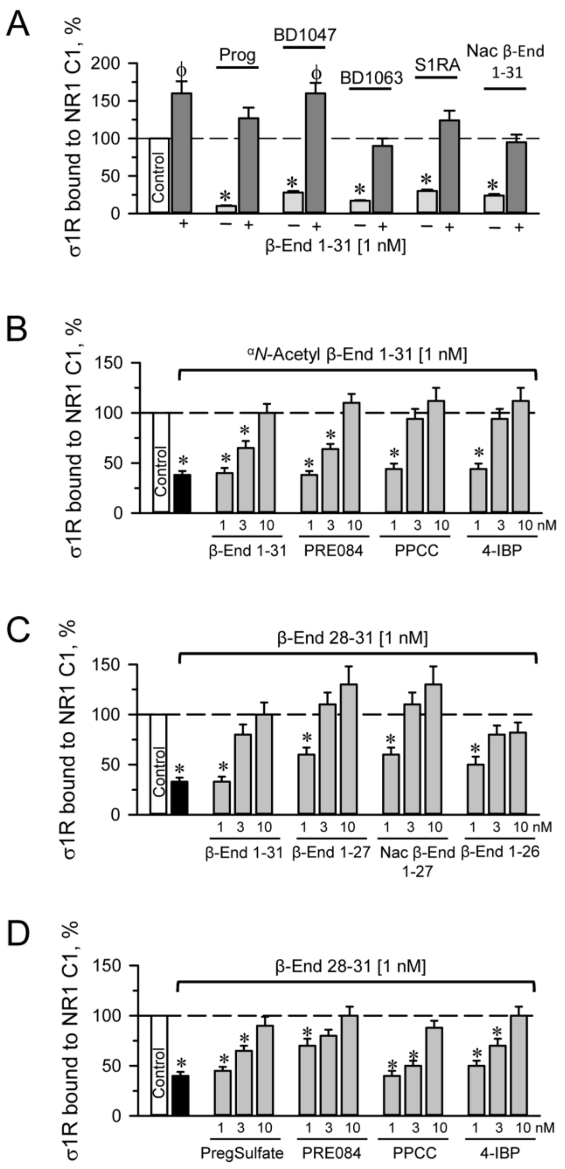

Figure S9. Effect of σ1R agonist/antagonist ligands on σ1R-NR1 C1 associations. Θ-Agarose-NR1 C1 was loaded with recombinant σ1Rs. After unbound σ1R proteins were removed, the complexes were incubated with **(A)** 1 nM β-End 1-31, **(B)** α*N*-acetyl β-End 1-31, **(C-D)** β-End 28-31 in the absence (black columns) and presence of **(A)** 10 nM or **(B-D)** increasing concentrations of competing σ1R ligands as indicated. The σ1Rs that remained attached to the Θ-σ2Rs were evaluated by Western blotting. The details are shown in Figures S6 and 6B. Prog is progesterone; PregSulfate is pregnenolone sulfate; Nac is α*N*-acetyl.

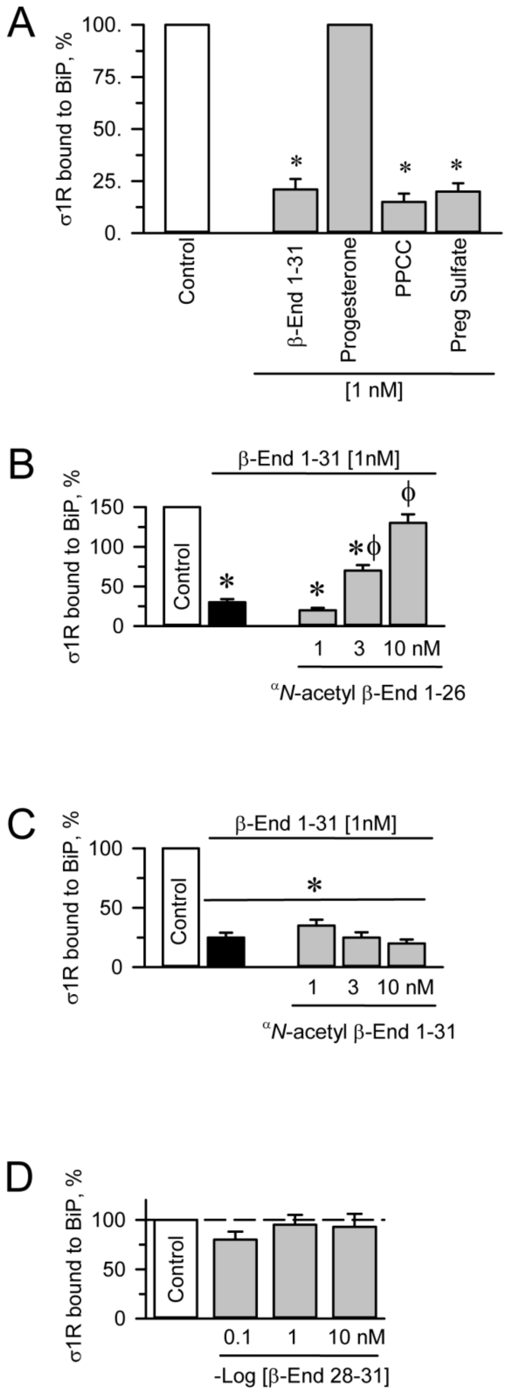

Figure S10. Effect of σ1R agonist/antagonist ligands on σ1R-BiP associations. Agarose-attached BiP proteins were incubated for 30 min in the presence of recombinant σ1R (100 nM) proteins in 300 μL of 50 mM Tris-HCl (pH 7.4), 0.2% CHAPS, and 2.5 mM $CaCl_2$. After the unbound σ1R proteins were removed, the effects of different σ1R ligands on BiP-σ1R associations were studied. The σ1Rs that remained attached to the agarose-BiP were evaluated by Western blotting. **A)** Effect of selected σ1R ligands (1 nM) on σ1R-BiP associations. ∗ Significant differences with respect to the control group incubated in the absence of ligands (white columns). PregSulfate is pregnenolone. **B-C**) Agarose-BiP-

σ1R complexes were incubated with 1 nM β-End 1-31 in the absence (black columns) or the presence of increasing concentrations of **(B)** $^{\alpha}$*N*-acetyl β-End 1-26, **(C)** $^{\alpha}$*N*-acetyl β-End 1-31 as indicated. * Significant differences with respect to the control group incubated in the absence of ligands (white columns). φ Significant difference with respect to the group that received only β-End 1-31 (black columns). ANOVA followed by the Holm-Sidak multiple comparisons test, $p < 0.05$. **D)** Lack of a β-End 28-31 effect on BiP-σ1R complexes. For details, see Methods and Figure 6C.

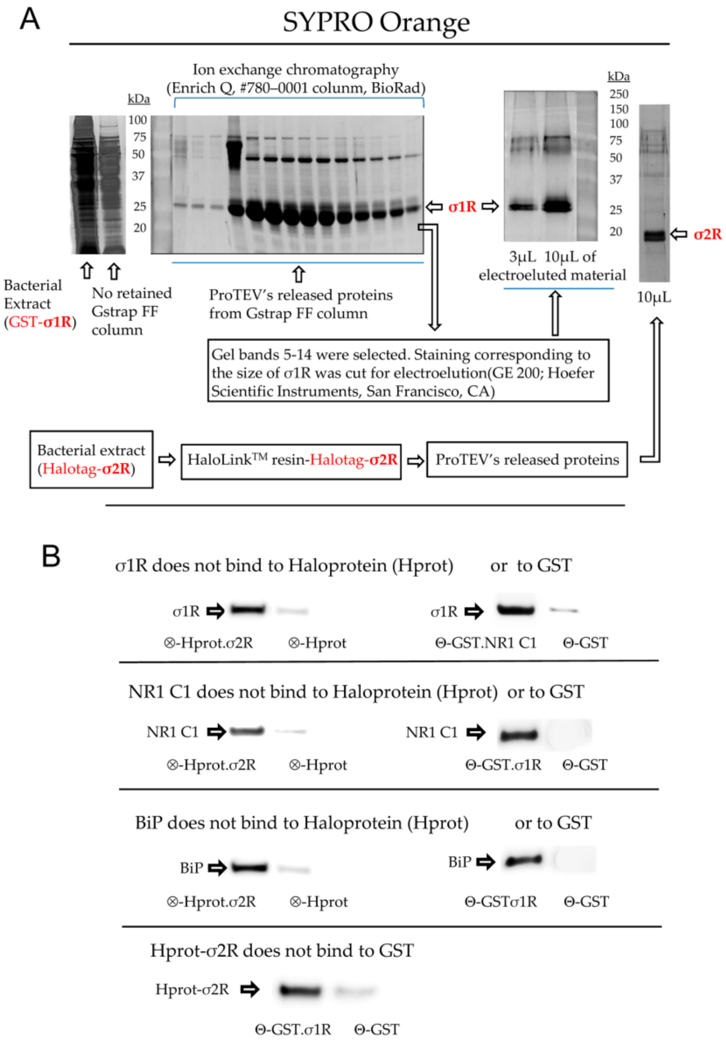

Figure S11. Purification of recombinants σ1R and σ2R proteins. Controls of σ1R1/2 interaction with Tag proteins GST and Haloprotein. **(A)** The proteins were stained using the SYPRO Orange Protein Gel Stain (#170-3120, BioRad). The purification procedure: briefly, specific primers containing an upstream Sgf I restriction site and a downstream Pme I restriction site were used. The RT-PCR products were cloned downstream of the GST/HaloTag® coding sequence (Flexi® Vector, Promega, Madrid, Spain) and the TEV protease site, and when sequenced, the proteins were identical to the GenBank™ sequences. The vector was introduced into the *E. coli* BL21 (KRX #L3002, Promega) and clones were selected on solid medium containing ampicillin. After 3 h of induction at room temperature (RT), in the presence of 1 mM isopropyl β-D-1-thiogalactopyranoside (IPTG) and 0.1% Rhamnose, the cells were collected by centrifugation and the pellets were maintained at -80 °C. The fusion proteins were purified under native conditions. The purification of GST fusion proteins was improved using SUMO1-agarose (#UL-740; Bos ton Biochem, Cambridge, MA), which binds the SIM domain of σ1R; this was followed by purification on GStrap FF columns (17-5130-01; GE Healthcare, Barcelona, Spain). The fusion proteins were cleaved on the column with ProTEV protease (#V605A; Promega) and the eluted material was injected in a high-resolution ion exchange (anionic) column (#780-0001Enrich Q, BioRad) using the

Biologic DuoFlow Chromatography system (4006229, BioRad). The proteins bound to the column were released using a continuous 0-1 M NaCl gradient. The fractions selected were concentrated in centrifugal filter devices (10,000-Da nominal molecular mass limit; Amicon Microcon YN-10, #42407 Millipore Iberica) and the medium was exchanged by Tris-HCl 50 mM pH 7,5, NaCl 50 mM, DTT 1 mM using PD-10 Desalting Columns (17-0851-01, GE Healthcare). Further purification was achieved by sodium dodecyl sulfate–polyacrylamide gel electrophoresis (SDS-PAGE) of the selected fractions. Gel bands were stained, those of interest removed and destained using the Copper Stain & Destain Kit for Electrophoresis (#161-0470, BioRad) and electroeluted (GE 200; Hoefer Scientific Instruments, San Francisco, CA). The Haloprotein-σ2R fusion proteins were purified under native conditions on HaloLink Resin (#G1915, Promega). The purity of the σ2R proteins was determined cleaving the HaloLink™-attached fusion proteins with ProTEV protease. When necessary this protein underwent the above described purification steps. Sequences were confirmed by automated capillary sequencing. The diffuse bands about 70-75 kDa accompanying the electroeluted proteins are produced by mercaptans added to the Laemmly buffer that react unspecifically with SYPRO.

(B), The recombinant proteins used in this study did not significantly bind to GST and Haloprotein (Hprot) which were used as tags. Approximately 100 nM of each σ1R, NR1 C1 and BiP proteins were incubated with HaloLink™-Hprot.σ2R / HaloLink™-Hprot, and with agarose-GST.NR1 C1 / agarose-GST. Hprot.σ2R was also incubated with agarose-GST-σ1R / agarose-GST. After 3 cycles of washing-resuspension to remove the unbound proteins, the samples were heated in a Laemmli buffer/β-mercaptoethanol, resolved by SDS-PAGE, transferred to PVDF blotting membranes and revealed with the corresponding antibodies. ⊗ HaloLink™ resin; Θ Agarose.
